# *Cuidando A Otros*: middle-aged and older Latinx-White caregiver disparities in financial distress and health strain in California

**DOI:** 10.1093/geroni/igaf137

**Published:** 2025-12-10

**Authors:** Alein Y Haro-Ramos, Julio Fernando Salas, Josefina Flores Morales

**Affiliations:** Department of Health, Society, and Behavior, University of California, Irvine, Joe C. Wen School of Population & Public Health, Irvine, California, United States; Department of Sociology, University of California, Berkeley, Berkeley, California, United States; Department of Epidemiology and Population Health, Stanford University, Palo Alto, California, United States

**Keywords:** Caregiving, Caregiver health strain, Racial/ethnic disparities, Latinx older adults

## Abstract

**Background and Objectives:**

Latinxs are a growing aging population in California and nationwide. However, limited research exists on middle-aged and older Latinx caregivers and the multidimensional toll of caregiving. This study examines Latinx–White disparities in 2 indicators of caregiving-related strain: financial distress and physical and mental health strain.

**Research Design and Methods:**

Using pooled cross-sectional data from the California Health Interview Survey (2019, 2020), we employ multivariable logistic regression models to examine financial distress associated with caregiving and caregiver-related health strain among Latinx and White adults aged 50 and older.

**Results:**

Latinx caregivers were more likely to live below the federal poverty level, be younger, report poor or fair health, be employed, and care for a family member than their White counterparts. Latinx caregivers had higher odds of reporting financial distress due to caregiving (odds ratio [OR]: 1.3, 95% CI, 1.06-1.59) than White caregivers; however, socioeconomic factors and caregiving dynamics explained this relationship. Compared to White caregivers, Latinx caregivers were less likely (OR: 0.60, 95% CI, 0.43-0.84) to report caregiver health strain, even in the fully-adjusted models and despite worse self-rated health.

**Discussion and Implications:**

Findings from this study underscore the need for targeted policies that alleviate the financial burden of caregiving for older Latinx adults, especially given their disproportionate exposure to economic precarity and high-intensity caregiving. While Latinx caregivers report lower caregiving-related health strain—potentially due to cultural resilience or social support—this should not obscure the structural disadvantages they face.

Innovation and Translational Significance: This study shows that middle-aged and older Latinx caregivers experience greater caregiving-related financial distress but lower caregiving health strain compared with White caregivers, despite worse overall self-rated health. Using population-based survey data, we identify underlying structural vulnerabilities, including poverty, continued labor force participation, and intensive caregiving responsibilities. These findings underscore the need for culturally tailored interventions and policies, including targeted financial support, paid leave, and accessible caregiver supports, to alleviate financial distress and support family caregivers. By addressing these caregiving challenges, this research provides a basis for strategies to strengthen family caregiving systems and reduce societal inequities in aging populations.

## Background and objectives

As the US population ages, 2 pressing concerns—health and financial security—dominate public discourse. Yet these conversations often overlook the unpaid infrastructure sustaining older adults’ daily lives: informal caregivers. Family members and other social ties often serve as “private safety nets,” absorbing the labor and costs of caring for older adults in unpaid, emotionally taxing, and financially destabilizing ways.^1^ Understanding caregiving patterns, particularly among structurally marginalized populations, is therefore a critical public health and policy concern.

Latinx adults are disproportionately represented among informal caregivers in the United States.^2^ Compared to non-Latinx White adults (hereafter referred to as White), Latinxs are more likely to provide care (21% vs 16.9%) and are overrepresented among high-intensity caregivers who spend more hours per week in caregiving roles.^2^ Although Latinx older adults have a longer life expectancy than their White counterparts, they tend to experience functional limitations at earlier ages^3^ and are more likely to reside in multigenerational households, which can extend caregiving responsibilities across generations.^4^ Latinxs also constitute both the fastest-growing aging population and the largest minoritized group in the United States,^5–7^ with the proportion of older Latinxs projected to rise from 8% to 21% in the coming decades.^8^ Growing caregiving experiences are shaped by intersecting cultural values (eg, familism, *respeto*),^9^ structural inequities (eg, lower lifetime earnings, limited employment benefits), and multigenerational family demands that together heighten financial strain and caregiver-related health challenges.

In this study, we aim to provide a population-based portrait of Latinx caregivers aged 50 and older in California, where Latinxs comprise about 40% of the population.^10^ Guided by life course theory (LCT) and the concept of *linked lives*, our aims are 2-fold: (1) Examine caregiving-related financial distress and the social and economic factors contributing to disparities between Latinx and White caregivers and (2) Assess whether disparities in caregiving-related health strain exist between middle-aged and older Latinx and White caregivers.

Together, these aims provide a more holistic understanding of how middle-aged and older Latinx adults navigate caregiving amid intersecting systems of aging, racialized inequality, and economic insecurity. We focus on caregivers aged 50 and older because caregiving dynamics and consequences differ substantially across the life course. While the average caregiver is approximately 49 years old,^11^ individuals in the 50+ age range are more likely to provide care while simultaneously managing their own age-related health changes, retirement transitions, and economic vulnerabilities. Further, despite their central role, middle-aged and older Latinx caregivers remain understudied, and examining this population allows for a nuanced understanding of how structural inequalities, cultural values, and aging-related vulnerabilities intersect to influence 2 key dimensions of caregiving-related strain: financial distress, reflecting the economic strain associated with caregiving responsibilities, and health strain, reflecting the physical and mental toll of care provision. These outcomes represent interconnected pathways through which caregiving can shape caregivers’ well-being.

### The economic resources of middle-aged and older Latinx adults

Caregiving imposes substantial financial demands across all racial and ethnic groups.^12^ Many caregivers experience economic strain through reduced employment opportunities, lost income, and out-of-pocket expenses (eg, transportation, medical supplies, home modifications).^13^ These pressures often intensify as caregiving increases in intensity and time commitment. For middle-aged and older adults nearing or past retirement, such trade-offs can jeopardize both current financial stability and long-term economic security.

For Latinx caregivers, these challenges unfold within a broader context of systemic economic disadvantage. Across the life course, Latinx individuals have faced lower wages,^14^ limited access to stable, pensioned employment,^15^^,^^16^ and discriminatory barriers in labor markets and financial systems—including wage theft,^17^ housing loan discrimination,^18^ and restricted access to wealth-building opportunities.^19^ Economic conditions experienced during young and middle adulthood may amplify into even more significant inequities in financial resources later in life.^20^ These cumulative inequities leave many Latinx caregivers with fewer economic reserves and weaker safety nets in later life.

Gender and nativity further compound these disadvantages. Latinx women and immigrants, who disproportionately serve as caregivers, are concentrated in low-wage, unstable jobs that offer limited benefits and few pathways to retirement security.^21^ As a result, when caregiving responsibilities arise, Latinx caregivers often must “do more with less”—absorbing caregiving costs and income losses within already constrained household budgets. In qualitative studies, Latinx families describe caregiving as both a moral obligation and a financial necessity, often stepping in because paid care options are unaffordable.^22^

These structural conditions shape not only the prevalence of caregiving among Latinx adults but may also influence the degree of financial distress associated with it. Given long-standing racialized inequalities in economic resources and employment opportunities, middle-aged and older Latinx caregivers may be more vulnerable to the financial consequences of unpaid caregiving than their White counterparts. Building on these insights, this study’s first aim is to assess Latinx–White disparities in caregiving-related financial distress and to identify the social and economic factors contributing to these differences. Our first hypothesis is:

***Hypothesis 1:*** *Middle-aged and older Latinx caregivers will report higher levels of financial distress compared with their White counterparts, and this relationship will be attenuated after accounting for pre-existing socioeconomic constraints.*

### Caregiving-related health strain

Along with financial distress, caregiving can impose significant physical and emotional strain, with caregivers often reporting higher levels of psychological distress, sleep disturbances, and chronic illness compared to non-caregivers.^23^^,^^24^ Yet, the relationship between caregiving and well-being is highly variable, shaped by factors, such as caregiving intensity, access to support, financial resources, and cultural appraisals of the caregiving role.^25–32^

For middle-aged and older Latinx caregivers, these dynamics are especially complex. Many navigate caregiving in the context of multigenerational households,^33^ where overlapping responsibilities for aging parents, spouses, and younger generations heighten stress and demand. These roles often unfold alongside their own aging-related health challenges, creating a dual burden of giving and needing care. Furthermore, systemic disadvantages—including lifetime exposure to physically demanding work, underinsurance, and limited access to health-promoting resources—combined with minimal institutional support, can further increase vulnerability to physical and mental health issues among caregivers.

At the same time, cultural values such as familism and *respeto* may buffer the psychological and emotional impact of caregiving.^9^ Evidence suggests that some Latinx caregivers report similar or even better health outcomes than their White counterparts, despite high caregiving demands and fewer economic resources, highlighting the potential protective role of cultural resilience.^30^ Among Latinxs, caregiving is often framed as a moral duty and source of purpose, reinforcing family interdependence and providing emotional rewards that can offset the strain of caregiving responsibilities.^34^^,^^35^ However, the protective role of familism is nuanced. Previous work found that Mexican-origin caregivers often described an idealized view of familism but relied on fewer family members than potentially available, indicating that cultural beliefs do not always translate into shared caregiving practices.^29^ While familism can moderate stress, Latinx caregivers may still experience substantial negative impacts from daily caregiving demands.^36^

Overall, the effects of familism on caregiver burden and mental health are complex and may not always offset strain.^28^ Specifically, prior research indicates that Latinx caregivers can experience both heightened well-being and greater emotional strain simultaneously, particularly when they are the primary caregiver or co-reside with the care recipient.^7^ This dual pattern suggests that the caregiving experience may involve both rewards and stress in ways that are not strictly linear.

These countervailing forces—structural vulnerability versus cultural buffering—make the expected direction of Latinx–White disparities in caregiving-related health strain uncertain. Middle-aged and older Latinx caregivers may be at higher risk due to cumulative disadvantage and limited access to formal supports, but cultural frames and intergenerational solidarity may mitigate the physical and mental toll of caregiving. Our second hypothesis is:

***Hypothesis 2****: Compared to White caregivers aged 50 and older, Latinx caregivers in the same age group will experience a different likelihood of caregiving health strain, defined as physical or mental health problems associated with caregiving.*

### Theoretical framework: life course theory, linked lives, and caregiving

To understand racial-ethnic disparities in caregiving-related financial distress and caregiving-related physical and mental health strain, this study draws on LCT. LCT offers a powerful lens for examining how caregiving experiences—and their financial and health strain consequences—are shaped by timing of life events and transitions, cumulative disadvantage, and interdependence of caregiving.^37^ A core tenet of this framework, *linked lives*, is especially relevant for examining how caregiving responsibilities and their impacts are embedded within families and social networks.^37^ This perspective highlights that disparities in caregiving outcomes are not merely individual phenomena but rather reflect interdependent lives shaped by enduring structural inequalities. Because the caregiving experience is deeply embedded in families and communities, rather than occurring in isolation, the tenet of *linked lives* underscores that caregiving decisions, burdens, and consequences are often distributed across a network of interdependent individuals.

To operationalize these concepts, we utilize variables that capture the relational, temporal, and structural dimensions of caregiving. Timing and transitions are represented by focusing on caregivers aged 50 and older, situating caregiving within a life stage marked by unique vulnerabilities and overlapping responsibilities, including health changes, retirement planning, and employment transitions. Cumulative disadvantage is captured through measures of poverty, education, and employment status, reflecting access to resources and structural constraints that influence caregiving experiences. Caregiving context variables—such as the relationship to the care recipient, co-residence, hours of care, and labor force changes due to caregiving—reflect the interdependent nature of caregiving. Together, these variables operationalize the core tenets of LCT, ensuring that our study captures how structural, cultural, and relational factors intersect to shape the experiences of middle-aged and older Latinx caregivers.

## Research design and methods

### Data and sample

Data for this analysis were derived from the 2019 and 2020 California Health Interview Survey (CHIS), a representative survey of community-dwelling adults and children in California. CHIS employs address- and cellphone-based sampling, with targeted oversampling, to ensure the inclusion of diverse populations. Participants completed questionnaires online or by phone in English, Spanish, Mandarin, Cantonese, Vietnamese, Korean, or Tagalog. In 2019, 22,160 adults completed CHIS, and 21,949 adults participated in 2020.

From the combined 44,109 adult respondents, we identified 11,518 individuals who provided care to a family member or friend with an illness or disability in the past year. Caregivers were identified with a positive response to the item: “*Some people provide short-term or long-term help to a family member or friend who has a serious or chronic illness or disability. This may include help with things they cannot do for themselves. During the past 12 months, did you provide any such help to a family member or friend?”* We then restricted the sample to self-identified Latinx and White caregivers aged 50 years and older, yielding a final analytic sample of 6895 respondents (5845 White and 1050 Latinx). Because the CHIS is a repeated cross-sectional survey, respondents from 2019 and 2020 represent distinct individuals.

### Measures

#### Outcome variables

The first outcome measure is financial distress due to caregiving. The following item assessed financial distress: “How much of a financial stress would you say that caring for [care recipient] was for you?” Respondents who selected “extremely” or “somewhat*”* stressful were classified as experiencing financial stress due to caregiving (coded as 1). In contrast, those who selected “a little” or “not at all” were coded as 0.

The second outcome measure is caregiving-related health strain. This item was assessed with the following: “During the past 12 months, have you suffered any physical or mental health problems yourself as a result of providing care to your [care recipient]?” Response items include yes (coded as 1) and no (coded as 0).

#### Independent variable

We use self-reported ethnicity, including Hispanic/Latinx and non-Hispanic White, to estimate group differences. The item asked, “Are you Latino or Hispanic?” Affirmative responses were coded as 1 and 0 otherwise.

### Covariates

#### Gender

Individuals self-reported their gender identity, which we code as woman or man.

#### Age

Self-reported age was grouped into the following 5-year categories: 50-54 (reference), 55-59, 60-64, 65-69, 70-74, 75-79, 80-84, and 85+.

#### Education

The survey asked respondents: “What is the highest grade of education you have completed and received credit for?” Based on their responses, we have the following educational groups: less than high school (reference), high school graduate or GED, some college, associate’s degree, and at least a bachelor’s degree.

#### Poverty

We use Federal Poverty levels (FPL) to assess household poverty. This measure accounts for the number of people in the home supported by the household income. The poverty level measures total annual income as a percentage of the FPL. For instance, individuals with a household income of 0%-99% of the FPL are in the highest poverty group. Poverty is a categorical variable, with the following 4 categories: 0%-99% of the FPL (reference), 100%-199% of the FPL, 200%-299% of the FPL, and 300% or more above the FPL.

#### Marital status

The survey asked respondents, “Are you now married, living with a partner in a marriage-like relationship, widowed, divorced, or never married?” We used a categorical measure of marital status, resulting in the following categories: married (reference), living with a partner, widowed, separated, divorced, and never married.

#### Rurality

We use a CHIS measure that classifies respondents’ place of residence in California as urban or rural.

#### Employment status

The survey asked, “What was your employment status in the last year?” Response items included: Full-time employment, part-time employment, other employed, unemployed, looking for work, and unemployed not looking for work. Those who responded full-time, part-time, or another employment category were classified as “Employed,” and those reporting otherwise were classified as “Not in the labor force.”

#### Labor force changes due to caregiving

We created a binary measure of labor force reduction based on a series of survey questions that assessed how caregiving affected respondents’ labor force participation. This measure was based on the following survey item: “Has your work situation changed because of helping your [care recipient], such as a change in job position, reduced number of work hours, quitting, or retiring?” Response options were nonexclusive and included (1) Changed job, (2) Took a second job/increased hours with current job, (3) Reduced the number of work hours, (4) Temporary leave of absence, (5) Quit job or got laid off, (6) Retired/retired early, (7) Received paid family leave. We created a measure indicating reduced work, temporary leave from work, quitting job, or getting laid off (response options (3), (4), or (5)). Our binary measure was coded as 1 if individuals experienced at least one of these types of reductions in work or 0 if none of these response options were endorsed.

#### Relationship to care recipient

Respondents were asked: “What is this person’s relationship to you?” We created a categorical measure of relationship ties based on a broader list of relationship ties in the survey: (1) spouse or partner, (2) parent or in-law, (3) other family ties (ie, sibling/sibling-in-law, grandparent, son/daughter, son- or daughter-in-law, another relative), and (4) friend/neighbor/other non-relative.

#### Caregiving time commitment

We also account for the number of hours dedicated to caregiving in a week. Based on the response distribution, we stratified by the median number of hours (5 hours per week). We categorized caregiving time commitment into 2 groups: 1-5 hours (reference group) and more than 5 hours per week.

#### Care recipients’ health conditions

The survey asked questions regarding the health conditions care recipients experienced. Respondents were asked, “At the time you provided care, what disabilities or illnesses does/did [care recipient] have that requires/required your help? Check all that apply (1) Alzheimer’s, confusion, dementia, forgetfulness; (2) arthritis; (3) back problems; (4) broken bones; (5) cancer; (6) diabetes, (7) feeble, unsteady, falling; (8) lung disease, emphysema, chronic obstructive pulmonary disease; (9) mental illness, emotional illness, depression; (10) mobility problem, can’t get around; (11) old age, aging; (12) stroke; and (13) surgery, wounds, or other. We created an index of the care recipient’s health conditions.

#### Other care recipients’ characteristics

We include a binary indicator assessing whether the care recipient lives with the caregiver (yes/no). Care recipient age was categorized into 3 groups (<65, 65-84, and 85+) to reflect key life course stages associated with differing care needs and caregiving demands. Adults under 65 include both younger and middle-aged recipients, who may have chronic illness or disability. In contrast, those aged 65-84 and 85+ represent older and oldest-old groups with increasing care intensity and complexity.

#### Caregivers’ health

We include a measure of global health. Respondents were asked to rate their health (“Would you say that in general your health is……?”) with 5 responses: “excellent,” “very good,” “good,” “fair,” and “poor.” “Fair” and “poor” categories were combined to represent adverse health, and all other responses were classified as “at least good health,” as has been done in previous health inequalities research.

### Analytic plan

We begin the analysis with descriptive distributions of the outcomes of interest and covariates stratified by Latinx and White caregivers aged 50 and older. We use *t*-tests for continuous variables and chi-squared tests for categorical variables.

We employed block-wise logistic regression models, in which variables were added sequentially in pre-specified blocks based on theoretical and conceptual relevance. Specifically, we began with an unadjusted model to examine the relationship between caregiver race/ethnicity and outcomes (Model 1), which is necessary for identifying baseline disparities. In Model 2, we adjusted for demographic characteristics (ie, caregivers’ gender, age, marital status, and rurality/urban). Model 3 incorporated socioeconomic variables (employment, education, and federal poverty level), reflecting social and structural resources that may partially explain observed disparities, as these factors constrain or facilitate access to caregiving resources. Model 4 included caregiving context variables (ie, care intensity, co-residence, care recipient characteristics, and caregiving-related labor force changes), which are most directly related to the outcomes and capture the embeddedness of caregiving within family networks, consistent with the *linked lives* principle. For the caregiving-related health strain analysis, we include an additional model adjusting for respondents’ self-rated health.

## Results

Table 1 reveals important differences in the demographic, caregiving, and care recipient characteristics of Latinx and White caregivers aged 50 and older in California. Latinx caregivers were younger on average, with higher representation in the 50-59 age group and fewer in the 70+ age bracket (*p *< .001), and were more likely to be employed (56.8% vs 48.3%, *p *< .001). Although Latinx caregivers were more likely to be women than men (62.2%), the difference in gender distribution compared to White caregivers (58.7%) was not statistically significant (*p *= .103). Latinx caregivers were significantly more likely to live in poverty, with 13.9% living below the federal poverty level compared to 5.6% of White caregivers (*p *< .001), and had substantially lower levels of educational attainment—only 18.6% held a Bachelor’s degree or higher, compared to over half (50.6%) of White caregivers (*p *< .001). However, there were no significant differences between Latinx and White caregivers in self-reported labor force disruptions due to caregiving (*p *= .204).

**Table 1.** Demographic characteristics of the sample, caregivers who are 50 y and older, by Latinx/non-Latinx White status; California Health Interview Survey, 2019, 2020.

Variable	White	Latinx	Total	*p*
**Unweighted *N***	5845	1050	6895	
***Caregiver characteristics***				
**Self-reported gender, %**				.103
** Male**	41.3	37.8	40.1	
** Female**	58.7	62.2	59.9	
**Poverty level, %**				<.001
** 0%-99% FPL**	5.6	13.9	8.4	
** 100%-199% FPL**	10.8	25.3	15.6	
** 200%-299% FPL**	12.7	16.7	14.0	
** 300% FPL and above**	70.8	44.2	62	
**Age, %**				<.001
** 50-54 y**	14.9	27.6	19.1	
** 55-59 y**	17.3	25.5	20.0	
** 60-64 y**	20.6	16.9	19.3	
** 65-69 y**	16.3	13.6	15.4	
** 70-74 y**	12.5	9.0	11.4	
** 75-79 y**	8.2	4.4	7.0	
** 80-84 y**	6.1	1.5	4.6	
** 85+ y**	4.0	1.5	3.2	
**Marital status: age 45 and older, %**				.9102
** Married**	63.6	65.2	64.1	
** Living with partner**	5.0	5.7	5.3	
** Widowed**	8.8	7.5	8.4	
** Divorced/separated**	15.3	14.4	15	
** Never married**	7.3	7.1	7.2	
**Education, %**				<.001
** Less than high school**	4.8	35.4	14.9	
** High school graduate/GED**	20.0	22.1	20.7	
** Some college**	18.4	18.5	18.4	
** Associate’s degree**	6.2	5.4	6.0	
** Bachelor’s degree and above**	50.6	18.6	40	
**Working status, %**				<.001
** Employed**	48.3n	56.8	51.1	
** Not in the labor force**	51.7	43.2	48.9	
**Caregiver self-rated health, %**				<.001
** At least good**	86.6	74.4	81.8	
** Poor/fair**	13.4	25.6	18.2	
***Caregiving characteristics***				
**Care recipient tie, %**				<.001
** Spouse**	20.5	17.4	19.5	
** Parents or in-laws**	40.3	45.7	42.1	
** Other relatives**	20.5	26.3	22.4	
** Friend, neighbor, or another nonrelative**	18.7	10.6	16.0	
**Care recipient age, %**				.001
** Younger than 65**	22.1	27.5	23.9	
** 65-84**	42.6	43.6	42.9	
** 85+**	35.3	28.9	33.2	
**Caregiving hours in a typical week, %**				.001
** 1-5 h**	49.1	41.5	46.6	
** >5 h**	50.9	58.5	53.4	
**Care recipient lives with caregiver**	37.9	46.3	40.7	.001
***Care recipient health conditions***				
** Chronic conditions (diabetes, cancer, lung disease), %**	26.7	31.9	28.4	.016
**Age-related condition (frailty/feeble/unsteady, old age, mobility problem, vision)**	57.1	56.1	57.6	.449
** Alzheimer’s disease, %e**	24.7	18.9	23.1	.001
** Neurological issues or stroke, %**	15.2	13.2	14.5	.194
** Musculoskeletal issues, %**	27.5	30.9	28.6	.143
** Mental health issues, %**	12.9	17.3	14.4	.016
** Other issue or medical condition, %**	18.6	16.1	17.8	.059
**Care recipient condition summary (mean [*SD*])**	1.84 [1.05]	1.89 [1.1]	1.84 [1.05]	.091
**Care Recipient Condition Index, %**				.353
** 1**	51.5	50.7	50.3	
** 2**	25.6	25.1	25.8	
** 3**	15.4	14.4	15.3	
** 4**	6.2	5.7	6.0	
** 5**	1.0	3.8	2.3	
** 6**	0.3	0.2	0.3	
** 7**	0	0.1	0	
**Reduced work, temporary leave, or quit/laid off, %**	8.4	10.1	8.9	.204
**Financial stress due to caregiving: Extremely or somewhat stressful, %**	17.7	23.4	19.6	.001
**Suffered mental health or physical health due to caregiving, %**	14.3	10.6	13.1	.032

Abbreviations: FPL = federal poverty level; GED = general education degree. Weighted statistics are shown. All column shows descriptive statistics for Latinx and White persons combined. Statistics sharing a letter are not statistically significantly different at the 5% level; for health conditions, each variable is tested separately; comparisons should be read row by row.

In terms of health, Latinx caregivers were significantly more likely to report fair or poor self-rated health compared to White caregivers (25.6% vs 13.4%, *p *< .001), despite their younger age profile. Caregiving dynamics also differed: Latinx caregivers were more likely to provide care to family members (parents/in-laws: 45.7% vs 40%, *p* < .001; other relatives: 26.3% vs 20.5%, *p *< .001), co-reside with the care recipient (46.3% vs 37.9%, *p *= .001), and deliver more than 5 hours of care per week (58.5% vs 50.9%, *p *= .001). They were also more likely to care for younger recipients (<65 years, *p *= .001).

Regarding care recipients’ health, Latinx caregivers more frequently reported that care recipients had chronic health conditions (31.9% vs 26.7%, *p *= .016) and mental health issues (17.3% vs 12.9%, *p *= .016), whereas White caregivers were more likely to care for someone with Alzheimer’s disease (24.7% vs 18.9%, *p *= .001). Finally, Latinx caregivers were more likely to report financial stress due to caregiving (23.4% vs 17.7%, *p *< .001), though they were significantly less likely to report caregiving-related mental or physical health strain (10.6% vs 14.3%, *p *= .032).

Table 2 presents odds ratios (ORs) estimating the likelihood of reporting financial distress due to caregiving. In Model 1, Latinx caregivers aged 50 and older had significantly higher odds of reporting financial distress due to their caregiving role compared to their White counterparts (OR = 1.42, 95% CI, 1.17-1.71). This disparity persisted after adjusting for demographic characteristics—age, gender, rurality, and marital status—in Model 2, though the association attenuated slightly (adjusted OR [aOR] = 1.30, 95% CI, 1.06-1.59). In Model 3, after further adjusting for caregivers’ socioeconomic characteristics, the association was no longer statistically significant (aOR = 1.06, 95% CI, 0.85-1.33). In the fully-adjusted Model 4, which additionally accounted for caregiving dynamics—such as care intensity, co-residence, care recipient characteristics, and caregiving-related labor force disruptions—the OR for Latinx caregivers further diminished and remained nonsignificant (aOR = 1.01, 95% CI, 0.8-1.28).

**Table 2.** ORs from a logistic regression estimating the extremely or somewhat extremely financially distressed due to caregiver status, California Health Interview Survey, 2019, 2020 (*n* = 6895).

Variable	(1)	(2)	(3)	(4)
	OR	OR	OR	OR
**Latinx**	1.42**	1.30*	1.06	1.01
	(1.17, 1.71)	(1.06, 1.59)	(0.85, 1.33)	(0.80, 1.28)
**Female (Ref. Male)**		1.26*	1.24*	1.09
		(1.05, 1.50)	(1.04, 1.48)	(0.88, 1.33)
**Age (Ref. 50-54 y old)**				
** 55-59 y old**		0.87	0.91	0.88
		(0.65, 1.18)	(0.67, 1.23)	(0.63, 1.23)
** 60-64 y old**		0.55**	0.58**	0.55**
		(0.42, 0.73)	(0.43, 0.77)	(0.39, 0.76)
** 65-69 y old**		0.46**	0.51**	0.60**
		(0.34, 0.62)	(0.37, 0.71)	(0.42, 0.84)
** 70-74 y old**		0.60**	ss0.68*	0.78
		(0.43, 0.83)	(0.46, 0.98)	(0.52, 1.17)
** 75-79 y old**		0.38**	0.42*	0.57
		(0.20, 0.71)	(0.22, 0.83)	(0.29, 1.14)
** 80-84 y old**		0.65	0.67	0.89
		(0.40, 1.05)	(0.39, 1.15)	(0.50, 1.57)
** 85+ y old**		1.04	1.08	1.27
		(0.46, 2.35)	(0.46, 2.56)	(0.52, 3.07)
**Marital status (Ref. Married)**				
** Living with a partner**		0.83	0.77	0.79
		(0.50, 1.37)	(0.48, 1.25)	(0.47, 1.32)
** Widowed, divorced, or separated**		0.96	0.83	0.92
		(0.76, 1.22)	(0.65, 1.06)	(0.70, 1.19)
** Never married**		0.86	0.76	0.80
		(0.55, 1.33)	(0.49, 1.19)	(0.49, 1.29)
**Rural (Ref. Urban)**		1.26	1.21	1.24
		(0.99, 1.60)	(0.96, 1.53)	(0.95, 1.61)
**Employed (Ref. Not in labor force)**			1.21	1.10
			(0.98, 1.49)	(0.87, 1.38)
**Education (Ref. less than high school)**				
** High school graduate/GED**			1.26	1.17
			(0.79, 2.02)	(0.73, 1.87)
** Some college**			1.03	1.00
			(0.67, 1.59)	(0.64, 1.57)
** Associate’s degree**			0.90	0.85
			(0.54, 1.49)	(0.49, 1.50)
** Bachelor’s degree and above**			0.93	0.99
			(0.60, 1.44)	(0.63, 1.56)
**Poverty-level (Ref. <100% FPL)**				
** 100%-199% FPL**			1.22	0.98
			(0.73, 2.04)	(0.58, 1.64)
** 200%-299% FPL**			0.84	0.71
			(0.51, 1.37)	(0.42, 1.20)
** 300% FPL and above**			0.60*	0.59*
			(0.38, 0.93)	(0.37, 0.94)
**Care recipient relationship (Ref. Partner)**				
** Parent or in-law**				1.59*
				(1.08, 2.36)
** Other relative**				1.09
				(0.75, 1.60)
** Non-relative**				0.83
				(0.48, 1.46)
**Caregiver reduced work, temp left workforce, quit, or was laid off (Ref. no)**				3.08**
				(2.35, 4.05)
**Care recipient age group** **(Ref. Care recipient is <64 y old)**				
** 65-84**				0.44**
				(0.33, 0.59)
** 85+**				0.44**
				(0.32, 0.62)
**>5 h/wk of caregiving (Ref. 1-5 h/wk of caregiving)**				2.02**
				(1.60, 2.55)
**Care recipient lives with caregiving** **(Ref. Care recipient lives elsewhere)**				1.97**
				(1.48, 2.60)
**Care Recipient Condition Index **				1.34**
				(1.22, 1.47)
**Constant**	0.21**	0.28**	0.35**	0.14**
	(0.19, 0.24)	(0.22, 0.35)	(0.18, 0.71)	(0.06, 0.35)

Abbreviations: FPL = Federal Poverty Line; GED = general education degree; OR = odds ratio. 95% confidence intervals are given in parentheses.

** *p *< .01,

* *p *< .05.

Other covariates also show noteworthy associations with financial distress. Caregivers who reduced work, temporarily left the workforce, or stopped working altogether had over 3 times the odds of experiencing financial distress. High-intensity caregiving (>5 hours/week), co-residence with the care recipient, and caring for individuals with multiple health conditions were also associated with greater financial distress. In contrast, caring for someone aged 65 or older was linked to lower odds of financial distress. While not specified a priori, these findings underscore the importance of care intensity and labor force disruptions in shaping caregivers’ financial vulnerability.

Table 3 presents results examining whether caregivers report mental or physical health problems due to caregiving, which we refer to as caregiver health strain. We present 4 models with the same covariates as in the previous analysis, plus an additional model that accounts for caregivers’ self-rated health. In Model 1, Latinx caregivers were less likely to report caregiver health strain (OR: 0.71, 95% CI, 0.54-0.93). This relationship remains statistically significant after adjusting for sociodemographics in Model 2 (aOR: 0.64, 95% CI, 0.48-0.85) and for socioeconomic position in Model 3 (aOR: 0.65, 95% CI, 0.47-0.90). In Model 4, including caregiving characteristics, the magnitude of the relationship slightly decreases, but Latinx caregivers still report lower odds of experiencing caregiver health strain (aOR: 0.60, 95% CI, 0.43-0.84). The association remains robust when adjusting for caregivers’ self-rated health in Model 5 (aOR: 0.58, 95% CI, 0.42-0.82).

**Table 3.** ORs from a logistic regression estimating whether caregiver suffered mental or physical health problems due to caregiving (*n* = 6895).

Variable	(1)	(2)	(3)	(4)	(5)
	OR	OR	OR	OR	OR
					
**Latino/x**	0.71*	0.64**	0.65**	0.60**	0.58**
	(0.54, 0.93)	(0.48, 0.85)	(0.47, 0.90)	(0.43, 0.84)	(0.42, 0.81)
**Female (Ref. Male)**		2.47**	2.46**	2.28**	2.30**
		(1.97, 3.10)	(1.97, 3.08)	(1.79, 2.91)	(1.80, 2.94)
**Age (Ref. 50-54 y old)**					
** 55-59 y old**		0.73*	0.71*	0.63**	0.61**
		(0.54, 0.99)	(0.52, 0.97)	(0.46, 0.87)	(0.44, 0.85)
** 60-64 y old**		0.84	0.78	0.68*	0.66*
		(0.61, 1.15)	(0.56, 1.07)	(0.48, 0.95)	(0.47, 0.93)
** 65-69 y old**		0.58**	0.52**	0.45**	0.45**
		(0.41, 0.82)	(0.36, 0.75)	(0.31, 0.67)	(0.30, 0.67)
** 70-74 y old**		0.60*	0.52**	0.44**	0.44**
		(0.40, 0.92)	(0.34, 0.80)	(0.28, 0.69)	(0.28, 0.69)
** 75-79 y old**		0.52**	0.46**	0.36**	0.38**
		(0.32, 0.84)	(0.28, 0.73)	(0.21, 0.61)	(0.22, 0.65)
** 80-84 y old**		0.40**	0.32**	0.22**	0.20**
		(0.21, 0.76)	(0.17, 0.64)	(0.10, 0.45)	(0.10, 0.43)
** 85+ y old**		0.77	0.64	0.40*	0.41*
		(0.32, 1.81)	(0.25, 1.60)	(0.18, 0.88)	(0.18, 0.95)
**Marital status (Ref. Married)**					
** Living with a partner**		0.74	0.72	0.71	0.68
		(0.43, 1.28)	(0.43, 1.23)	(0.40, 1.25)	(0.39, 1.21)
** Widowed, divorced, or separated**		1.51**	1.37**	1.65**	1.57**
		(1.21, 1.89)	(1.08, 1.73)	(1.28, 2.13)	(1.21, 2.04)
** Never married**		0.98	0.92	1.12	1.06
		(0.65, 1.48)	(0.62, 1.38)	(0.74, 1.71)	(0.70, 1.62)
**Rural (Ref. Urban)**		1.05	1.01	0.94	0.97
		(0.84, 1.32)	(0.81, 1.27)	(0.74, 1.20)	(0.76, 1.24)
**Employed (Ref. Unemployed)**			0.78**	0.70**	0.75*
			(0.64, 0.94)	(0.54, 0.89)	(0.58, 0.96)
**Education (Ref. less than High School)**					
** High school graduate/GED**			1.27	1.30	1.54
			(0.78, 2.05)	(0.81, 2.09)	(0.95, 2.50)
** Some college**			1.60*	1.75*	2.10**
			(1.05, 2.45)	(1.12, 2.74)	(1.33, 3.31)
** Associate’s degree**			1.62	1.77	2.09*
			(0.94, 2.81)	(0.99, 3.16)	(1.14, 3.84)
** Bachelor’s degree and above**			1.63*	1.98**	2.50**
			(1.09, 2.45)	(1.30, 3.01)	(1.62, 3.86)
**Poverty-level** **(Ref. <100% FPL)**					
** 100%-199% FPL**			1.39	1.12	1.23
			(0.90, 2.13)	(0.67, 1.86)	(0.72, 2.10)
** 200%-299% FPL**			0.88	0.82	0.99
			(0.50, 1.55)	(0.46, 1.47)	(0.54, 1.80)
** 300% FPL and above**			0.75	0.81	1.00
			(0.47, 1.20)	(0.48, 1.37)	(0.57, 1.73)
**Care recipient relationship (Ref. Partner)**					
** Parent or in-law**				0.55**	0.55**
				(0.36, 0.82)	(0.37, 0.83)
** Other relative**				1.03	1.06
				(0.74, 1.41)	(0.77, 1.46)
** Non-relative**				0.57*	0.55*
				(0.34, 0.95)	(0.33, 0.93)
**Caregiver reduced work, temp left workforce, quit, or was laid off (Ref. no)**				2.46**(1.78, 3.40)	2.58**(1.87, 3.55)
**Caregiver age group (Ref. Care recipient is <64 y old) **					
**65-84**				0.76	0.78
				(0.55, 1.04)	(0.57, 1.06)
**85+**				0.95	0.99
				(0.65, 1.38)	(0.69, 1.44)
**1-5 h/wk of caregiving** **(Ref. <5 h/wk of caregiving)**				1.67**(1.30, 2.14)	1.70**(1.33, 2.17)
**Care recipient lives with caregiver** **(Ref. lives elsewhere) **				1.55**(1.40, 1.72)	1.53**(1.38, 1.69)
**Poor/fair self-rated health**					2.36**
					(1.79, 3.11)
**Care Recipient Condition Index**				1.55**	1.53**
				(1.40, 1.72)	(1.38, 1.69)
**Constant**	0.17**	0.12**	0.12**	0.04**	0.02**
	(0.15, 0.19)	(0.09, 0.16)	(0.07, 0.20)	(0.02, 0.08)	(0.01, 0.05)

Abbreviations: FPL = Federal Poverty Line; OR = odds ratio. 95% confidence intervals are given in parentheses.

** *p *< .01,

* *p *< .05.

## Discussion and implications

In this study, we examine the association between being a Latinx older adult caregiver and financial stress and caregiver-related physical/mental health strain as critical dimensions of caregiving burden. In addition, we provide a detailed demographic portrait of Latinx caregivers 50 years and older in California.

First, we found critical demographic patterns among Latinx and White caregivers. Middle-aged and older Latinx caregivers were more likely to care for a proximal familial tie, including parents/in-laws, and also extended family ties, than their White counterparts. This finding may be attributed to the higher prevalence of multigenerational households among Latinx families. Latinx families comprise nearly 30% of the multigenerational households in the United States.^38^ In comparison, only 13% of White families live in a multigenerational household, making them the group least likely to do so in the United States.

We found no statistically significant differences in caregivers’ changes to labor force participation, including quitting or being laid off, taking temporary leave, or reducing work hours. Although this could be seen as a potentially positive factor, the explanation may be more complex. First, at baseline, White caregivers were more likely to report not being in the labor force, limiting the potential for measurable changes in their employment status. Second, supplemental analyses showed that Latinx caregivers in our sample had lower retirement rates due to caregiving than their White counterparts (7.48% vs 10.85%, see Supplementary Table 1), suggesting a stronger labor force attachment. This likely reflects economic necessity: Latinx caregivers, who face greater economic precarity, may be unable to afford time away from work. National data support this, showing that Latinx caregivers spend a larger share of their income on caregiving—47% compared to 18% among White caregivers^13^—highlighting the financial pressures that may constrain their labor force flexibility.

In support of *Hypothesis 1*, we found that middle-aged and older Latinx adults had a higher likelihood of reporting financial distress due to caregiving. However, when we accounted for socioeconomic factors and caregiving dynamics, the relationship decreased in magnitude and became statistically insignificant. This pattern suggests that the observed disparity in financial distress may be explained by pre-existing socioeconomic conditions and caregiving contexts in which Latinx adults are embedded. From a life course perspective, these findings reflect cumulative disadvantage—a process through which long-standing inequities in income, employment, and access to benefits compound over time and shape the resources available to manage later-life caregiving. In our sample, Latinx caregivers were more likely to live below the FPL despite higher rates of workforce participation than their White counterparts, underscoring how caregiving unfolds against a backdrop of structural economic vulnerability.

The principle of *linked* lives further illuminates these patterns. Caregiving in later life rarely occurs in isolation; rather, it is embedded in interdependent family systems where financial, emotional, and time resources are shared across generations. In this study, a higher share of Latinx caregivers provided care for parents or in-laws—relationships that often demand more time- and effort-intensive support. Given the higher prevalence of multigenerational households among Latinx families,^38^ caregiving can redistribute household labor and financial resources in ways that heighten financial strain. When older family members who once contributed economically become care recipients, caregivers often absorb a greater share of the household’s financial responsibilities. This interdependence may help explain why the Latinx–White disparity in financial distress diminishes once socioeconomic context and caregiving relationships are considered.

Finally, in *Hypothesis 2*, we posited that Latinx–White disparities in caregiving-related health strain would differ, with the direction of disparity theoretically ambiguous due to competing structural and cultural influences. Our findings revealed that middle-aged and older Latinx adults were *less likely* than their White counterparts to report caregiving-related physical or mental health strain. Our findings align with prior research suggesting that Latinx caregivers, despite facing greater economic and structural barriers, often report more favorable caregiving outcomes than expected.^28^^,^^34^^,^^39^

Several potential explanations may help reconcile this apparent contradiction. First, cultural values such as *familism*—a strong sense of obligation and emotional commitment to family—may foster positive meaning-making and psychological resilience in the caregiving role.^9^ Middle-aged and older Latinx caregivers may perceive caregiving not as a burden, but as a familial responsibility that carries social and moral significance, thereby reducing the likelihood of registering the role as physically or mentally taxing.

The tenet of *linked lives* also provides an interpretive lens for understanding why caregiving may not uniformly translate into health strain among middle-aged and older Latinx caregivers. Because caregiving is embedded in dense networks of reciprocal support, the emotional and social benefits derived from these interdependent relationships may offset some of the physical or mental costs of care. At the same time, our findings may also be partially explained by differences in health appraisal or reporting behavior across racial-ethnic groups. Latinx caregivers may underreport caregiving health strain due to stigma, cultural norms around stoicism, or differing thresholds for what constitutes health decline.

However, the lower prevalence of reported caregiving health strain should not be misinterpreted as evidence of a lower need for support among Latinx caregivers. Prior work has shown that caregiving strain can be underrecognized or internalized in communities where formal services are less accessible, and caregiving norms are deeply entrenched.^40^ Our findings underscore the importance of culturally attuned policies and services that account for both the subjective experience of caregiving and the structural conditions in which it occurs. The apparent protective health effects observed among middle-aged and older Latinx caregivers may reflect not only current cultural frames but also a lifetime of adaptive strategies developed in the context of structural exclusion and communal survival.

This research holds critical policy implications and may suggest the need for greater use and awareness of paid caregiver programs, especially amid a presidential administration that aims to eliminate this form of support.^41^ Given that 90% of older adults in the United States prefer to age in place rather than in an institution, paid caregiving programs could facilitate this goal. Aging in place has been associated with better perceived psychological and physical benefits among older adults.^42^ Bolstering the In-Home Supportive Services program in California—which provides financial compensation to caregivers who provide in-home care to older adults or people with disabilities—is one way to enable more individuals to age in place. However, increasing funding alone is not sufficient. Policymakers and practitioners must also address implementation barriers, including language access, complex application processes, and limited program awareness, to ensure equitable uptake among Latinx caregivers, who may experience greater financial and structural constraints than White caregivers. By targeting these barriers, supportive programs can help mitigate caregiving-related financial distress and reduce health disparities across racial-ethnic groups.

This study has several limitations. First, the cross-sectional design limits our ability to infer causality in our associations. Future research should address these limitations through longitudinal studies and by including more diverse populations to provide a more comprehensive understanding of the relationship between multidimensional aspects of caregiving strain among middle-aged and older caregivers. Second, we did not focus on differences by nativity or citizenship status. We encourage future researchers to examine how citizenship status shapes who Latinx caregivers care for, how long they care for, and their financial distress due to caregiving. Next, more qualitative research is needed to better elucidate why Latinx caregivers do not leave the workforce and how that shapes their caregiving responsibilities and their financial distress. Finally, we focused on caregivers aged 50 and older, limiting the generalizability of our findings. Younger caregivers may face different caregiving dynamics, financial challenges, and health impacts. Future research could compare the experiences of younger, middle-aged, and older caregivers.

Despite its limitations, this study has significant strengths. First, it used a broad, representative sample of older Latinx and White adults in California, home to the nation’s largest Latinx population. This enhances the likelihood that findings generalize to other states with significant numbers of Latinx caregivers. Second, the research highlights an often-overlooked demographic—Latinx caregivers entering middle and older adulthood. Latinxs are a diverse population, and their experiences of caregiving in older adulthood may be shaped by numerous social forces, including immigration, gender, family, and socioeconomic stratification. Understanding these complexities, especially the transnational aging phenomenon, will remain critical to understanding the ramifications of being a middle-aged and older Latinx caregiver.

## Conclusion

Our results provide a detailed portrait of middle-aged and older Latinx caregivers in California. Latinx caregivers were more likely than their White counterparts to report financial distress related to caregiving; however, this disparity was largely explained by differences in socioeconomic position and caregiving dynamics. Many Latinx caregivers in our sample remained employed yet lived below the federal poverty level, reflecting long-standing structural barriers to wealth accumulation, equitable wages, and access to benefits. These findings support the life course principle of *cumulative disadvantage*, showing how unequal access to economic resources earlier in life compounds into financial vulnerability during later life when responsibilities like caregiving become more prevalent. Latinx caregivers, however, reported less caregiving-related health strain than White caregivers, despite facing greater structural and health-related disadvantages. From a life course perspective, this apparent paradox may reflect the principle of *linked lives*—the deep interdependence and reciprocal care within multigenerational Latinx families—which may provide emotional and social resources that buffer against some forms of stress. However, these cultural dynamics should not be interpreted as offsetting or compensating for systemic inequities. Rather, they operate within and often in response to enduring structural disadvantage, where the reliance on family networks reflects limited access to institutional and economic supports for caregiving. Future research and policy efforts should address the structural foundations of caregiving inequality—such as labor protections, retirement security, and access to paid leave—while recognizing that the strength of family ties, though vital, cannot substitute for equitable economic and institutional supports.

## Supplementary Material

igaf137_Supplementary_Data

## Data Availability

Data are publicly available for use through the California Health Interview Survey. The study was not pre-registered.
