# Supplementary material for: Cuidando A Otros: middle-aged and older Latinx-White caregiver disparities in financial distress and health strain in California
Source: Innov Aging. 2025 Dec 10;10(3):igaf137. doi: 10.1093/geroni/igaf137 (PMC12924875; doi:10.1093/geroni/igaf137)
Supplement: igaf137_Supplementary_Data [file igaf137_supplementary_data.zip › innage suppl Haro-Ramos, Salas, & Flores Morales.docx]

***Innovation in Aging* Supplementary Material: Haro-Ramos, Salas, & Flores Morales. *Cuidando A Otros*: Older Latinx Caregiver Disparities, Financial Distress, and Health in California.**

**Supplementary Table 1.** Outcomes in employment transitions by Latinx-White status

|  | **Non-Latinx White** | **Latinx** | **All** | ***p*** |
| --- | --- | --- | --- | --- |
| Retired early/retiree | 11.19% | 7.57% | 10.01% | 0.006 |
| Laid off or quit | 1.74% | 3.13% | 2.20% | 0.039 |
| Temporary leave | 1.75% ^A^ | 2.07% ^A^ | 1.85% | 0.597 |
| Reduced work hours | 5.17% ^A^ | 5.96% ^A^ | 5.43% | 0.435 |
| Experienced a job change | 0.83% | 2.14% | 1.26% | 0.006 |
|  |  |  |  |  |
| Note: Shared letter (row-wise) means there is not statistically significant differences between Latinx and non-Latinx white respondents. | | | | |
